# Excessive disulfide bonds in lamin A/C contribute to premature human aging

**DOI:** 10.1016/j.mocell.2026.100389

**Published:** 2026-08-17

**Authors:** Seokjun G. Ha, Minho Park, Jinwook Lee, Dajeong Bong, Jinsook Ahn, Doyeon Kim, Myung-Ok Kim, Seung-Jae V. Lee, Nam-Chul Ha

**Affiliations:** 1 Department of Biological Sciences, Korea Advanced institute of Science and Technology, Daejeon 34141, South Korea; 2 Research Institute of Agriculture and Life Sciences, Center for Food and Bioconvergence, Department of Agricultural Biotechnology, CALS, Seoul National University, Seoul 08826, Republic of Korea

**Keywords:** LMNA mutation, immunoglobulin-like domain, progeroid laminopathy, nuclear deformation, oxidative stress

## Abstract

Nuclear lamins provide structural integrity to the nuclear envelope through coiled-coil dimer meshworks. Lamin A contains a C-terminal immunoglobulin (Ig)-like domain and a cysteine-rich unstructured tail, whereas lamin C lacks the latter, retaining only 1 cysteine within the Ig-like domain. Mutations R435C and R471C in the Ig-like domain are linked to progeroid syndromes, fatal disorders characterized by premature aging. Here, we elucidate a pathogenic mechanism driven by aberrant disulfide cross-linking. We found that the R435C mutation, but not R471C, facilitates successive disulfide bond formation between Ig-like domains in vitro using purified recombinant proteins, causing nuclear deformation in lamin C-overexpressing cells. In lamin A-overexpressing cells, both R435C and R471C mutations induce additional intermolecular disulfide bonds involving the lamin A-specific cysteine residues in the C-terminal tail. Importantly, we demonstrate that glutathione and its precursor, N-acetyl cysteine, can disrupt these aberrant bonds. Using *Caenorhabditis elegans* as an *in vivo* model, we show that the orthologous cysteine mutation causes progeria phenotypes, which are suppressed by antioxidant treatment. These findings identify aberrant disulfide cross-linking as a key driver of progeria and suggest antioxidant therapies as a potential treatment strategy. Our study offers broader implications for vertebrate aging, suggesting that oxidative stress-mediated changes in lamin architecture are a conserved mechanism contributing to the loss of nuclear structural integrity and age-dependent nuclear aberration.

## Graphical Abstract

**Figure:**
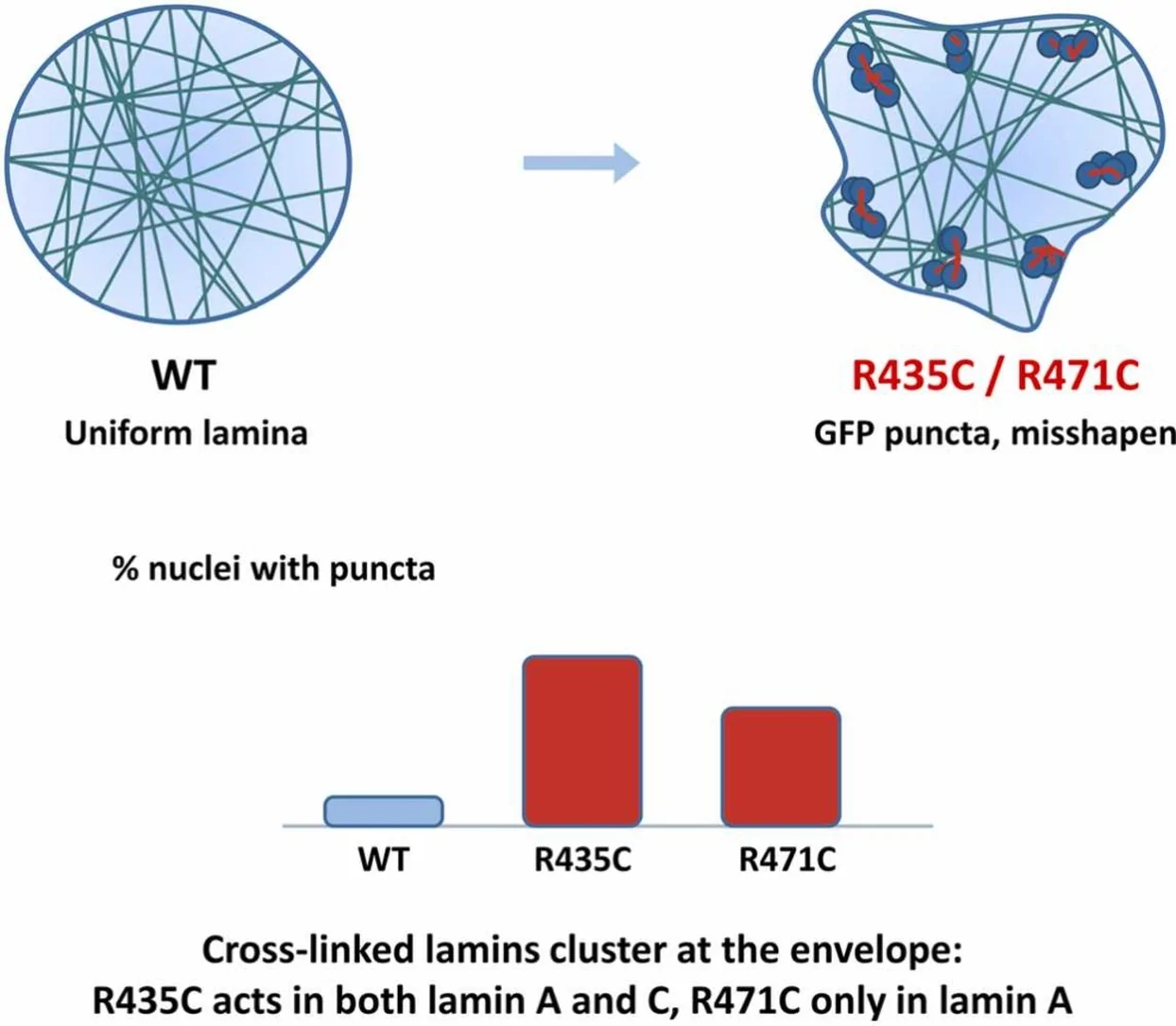

## BACKGROUND

Most vertebrate cells contain a nucleus that segregates and protects chromatin and other nucleoplasmic components from the cytoplasm and express nuclear lamins to maintain the structural integrity of the nuclear envelope (Coffinier et al., 2011, Kim et al., 2011). Lamins A/C, B1, and B2 are expressed in human cells and contribute to differential strength of nuclear envelopes. Lamins A and C, encoded by the *LMNA* gene in humans, are generated as alternative splicing products and provide structural support to the basal meshwork of lamins B1 and B2 (Lin and Worman, 1993, Röber et al., 1989, Shimi et al., 2008). Lamin A is expressed in muscle and blood vascular endothelial cells that undergo continual mechanical stress (Röber et al., 1989, Vlcek and Foisner, 2007). Lamin C is expressed more ubiquitously than lamin A; neuronal cells express only lamin C but not lamin A.

Lamins are type V intermediate filaments, consisting of a short N-terminal head, central ⍺-helical rod domain, and C-terminal tail domain. The central rod domain forms a parallel coiled-coil dimer, which acts as the fundamental unit for creating higher assembly structures of the laminar linear filament whose crystal structures are available (Ahn et al., 2019). The C-terminal tail domain of lamins is further divided into a flanking globular immunoglobulin (Ig)-like domain and an unstructured region containing the C-terminus. Lamin C has a shorter C-terminal tail downstream of the Ig-like domain (Machiels et al., 1996). The longer C-terminal unstructured region of lamin A is further processed by farnesylation and cleavage by the Zmpste24 endopeptidase, resulting in the maturation of lamin A (Pendás et al., 2002).

The Ig-like domains (residues 428-549) of lamins A and C exhibit an Ig fold, as revealed by their determined 3-dimensional structures (Ahn et al., 2021, Krimm et al., 2002). Although the functions of the Ig-like domain remain unclear, several laminopathies such as premature aging, muscular dystrophy, and lipodystrophy are triggered by point mutations within the Ig-like domain (Krimm et al., 2002). Notably, lamins A and C contain limited numbers of cysteine residues. Neither lamin A nor C proteins have a cysteine residue in the N-terminal head (residues 1-28) and central rod domain (residues 29-385). Lamins A and C both possess a single cysteine residue at Cys522 within the Ig-like domain, whereas lamin A also contains 4 additional cysteine residues in the longer C-terminal unstructured region (residues 549-664), distinguishing it from lamin C. Notably, the aberrant introduction of cysteine residues into Ig-like domains, as observed in certain mutations, is associated with the onset of progeroid syndromes (Madej-Pilarczyk et al., 2009), thus emphasizing the critical role of cysteine placement and modification in the pathology of premature aging diseases.

The genetic mutation R435C in the Ig-like domain results in progeroid laminopathy, with features resembling restrictive dermopathy (Starke et al., 2013). In normal human fibroblasts transfected with the R435C *LMNA* mutant, a significantly lower number of DNA repair foci was observed following H_2_O_2_-induced DNA damage compared with the wild-type *LMNA*-transfected cells. The R471C mutation causes a related but distinct recessive phenotype, a mandibuloacral dysplasia-progeria overlap with lipodystrophy and rigid spine muscular dystrophy, reported in the homozygous or compound-heterozygous state (Cao and Hegele, 2003, Zirn et al., 2008), and likewise impairs the formation of DNA repair foci when the mutant *LMNA* gene is expressed (Kwon et al., 2023, Manju et al., 2006). Both are very rare variants, and their pathogenicity in the homozygous state highlights the critical role of aberrant cysteine introduction into the Ig-like domain. However, the molecular roles of the cysteine residues in the mutant Ig-like domain remain unclear.

In this study, we examine the molecular effects of the progeroid syndrome-causing R435C and R471C mutations on lamins A and C. Our results show that these mutations introduce additional disulfide bonds, particularly in the lamin A-specific C-terminal region, leading to nuclear deformation in cells overexpressing mutant lamin C. We also demonstrate that glutathione (GSH) or its precursor N-acetyl cysteine can reverse these effects. Further, using the roundworm *Caenorhabditis elegans*, an established animal model for organismal aging research, we show that the orthologous cysteine mutation in lamin A causes progeria-like phenotypes, which are suppressed by antioxidant treatment. These findings suggest that disulfide bonds from cysteine residues in lamins play a key role in progeria and aging-related diseases.

## MATERIALS AND METHODS

### Plasmid Construction

Amplified DNA fragments encoding the lamin A/C short Ig-like domain (residues 406-552) and extended Ig-like domain (residues 406-570) were cloned into the pProEx-HTa vector (Thermo Fisher Scientific, Waltham, MA, USA). For protein tagging, the EZ-Fusion HT Cloning Kit (Enzynomics, Daejeon, Korea) was used to introduce eGFP and FLAG tags into the pCDNA3.1 vector (Invitrogen, Waltham, MA, USA). DNA encoding full-length lamin A and lamin C was subsequently inserted into pCDNA3.1-GFP and pCDNA3.1-FLAG, respectively. The point mutations R435C and R471C were introduced using polymerase chain reaction.

For *C elegans* LMN-1 protein expression, amplified DNA fragments encoding the Ig-like domain (residues 438-551) of the wild type and the E442C,I527C double mutant were cloned into the pProEx-HTa vector (Thermo Fisher Scientific, Waltham, MA, USA).

### Protein Overexpression and Purification

The plasmids encoding the short Ig-like domain (residues 406-552) and extended Ig-like domain (residues 406-570), as well as their respective mutants, were transformed into the *Escherichia coli* strain B834(DE3) (Novagen, Madison, WI, USA) for protein overexpression. The *C elegans* LMN-1 Ig-like domain (residues 438-551) and its mutant were transformed into *E coli* strain BL21(DE3). Cells were cultured in Luria-Bertani (LB) medium containing 50 μg/mL ampicillin at 37°C until the optical density at 600 nm (OD_600_) reached 0.6. Protein expression was induced by adding 0.5 mM isopropyl β-D-1-thiogalactopyranoside and incubating the cells at 30°C for 6 hours. The cells were harvested and resuspended in lysis buffer containing 20 mM Tris-HCl (pH 8.0), 150 mM NaCl, and 2 mM 2-mercaptoethanol. Cells were lysed using a continuous-flow French press (Constant Systems Limited, Daventry, UK) at 23 kpsi, followed by centrifugation to remove cell debris. The supernatant was loaded onto a Ni-NTA affinity resin (Qiagen, The Netherlands), and the protein was eluted with lysis buffer supplemented with 250 mM imidazole. The protein was further processed using recombinant tobacco etch virus protease to cleave the hexahistidine tag. The tag-free protein was subsequently purified using a linear gradient of 1M NaCl on a HiTrap Q column (GE Healthcare), followed by SEC using a HiLoad 16/600 Superdex 200 pg column (GE Healthcare) equilibrated with 20 mM Tris-HCl (pH 8.0) containing 150 mM NaCl. The purified proteins were concentrated to 20 mg/mL and stored at −80°C.

### Crystallization and Structure Determination

The Lamin A/C Ig-like domain R435C (17 mg/mL) with 5 mM DTT was crystallized and processed as previously described with minor modifications (Ahn et al., 2022). Precipitation solution containing 0.1 M sodium citrate tribasic dihydrate (pH 5.0) and 30% (v/v) Jeffamine ED-2001 (pH 7.0) was used. The crystals belonged to space group P6_4_22 with unit-cell dimensions of *a* = 92.0 Å, *b* = 92.0 Å, and *c* = 204.8 Å (Table S1). Data collection and processing details are presented in Table S1.

### Oxidative Stress Response of Proteins to Hydrogen Peroxide

Proteins from each mutant were diluted to a concentration of 1 mg/mL. Human lamin A/C proteins were treated with 0 to 1 H_2_O_2_, whereas *C elegans* LMN-1 proteins were treated with 0 to 10 mM H_2_O_2_. The samples were then incubated at 25°C for 30 minutes. To stop the reaction, 5 mM iodoacetamide (IAA) was added. Subsequently, 6X Laemmli loading buffer was added, and the samples were heated at 100°C for 5 minutes. Each sample (10 µL) was then loaded onto SDS-PAGE gels under both reducing and nonreducing conditions, which were established by the presence or absence of 10 mM DTT, respectively. The gels were stained with Coomassie Brilliant Blue for visualization.

### Transmission Electron Microscopy (TEM) Analysis

The short Ig-like domain protein (residues 406-552) was fractionated through SEC using a HiLoad 16/600 Superdex 200 pg column (GE Healthcare) after treatment with 1 H_2_O_2_ and incubated at room temperature for 30 minutes at a concentration of 20 mg/mL. Each fraction was diluted 1:100 and 10 μL was applied to a carbon-coated 400-mesh copper grid, which had been freshly glow-discharged. After incubation for 1 minute, the grid was washed thrice with 100 μL of distilled water. Subsequently, the grid was stained with 1% (w/v) uranyl acetate for 1 minute, blotted dry with a filter paper, and air-dried. Images were captured using an energy-filtering TEM, Carl Zeiss LIBRA 120 (Carl Zeiss, Oberkochen, Germany), at an accelerating voltage of 120 kV.

### Predictive Modeling of Lamin A/C Structures With AlphaFold2

The AlphaFold2 program (Akdel et al., 2022, Jumper et al., 2021, Varadi et al., 2022) was installed on a computer equipped with NVIDIA RTX 4090 (24 GB RAM) and 128 GB RAM, supporting 32 threads. Using AlphaFold2, predictive models for the tetrameric, pentameric, hexameric, and heptameric structures of the Ig-like domain of lamin A/C (residues 406-522) were generated. Model validation was performed using ALPHAPICKLE (Arnold, 2021) to analyze the pAE and pLDDT for Cα atoms.

### Culture and Transfection of HEK293E and A549 Cells

HEK293E and A549 cells obtained from the Korean Cell Line Bank (KCLB, Seoul, Republic of Korea) were cultured in Dulbecco’s modified Eagle’s medium (DMEM) supplemented with 10% fetal bovine serum (FBS) and 1% penicillin-streptomycin. Cultures were maintained at 37°C in a 5% CO_2_ atmosphere. After removing FBS, the cells were transfected with vectors specific to each experiment using Lipofectamine 3000 (Thermo Scientific, Waltham, MA, USA), according to the manufacturer’s protocol. Following transfection, the medium was replaced with fresh culture medium after 12 hours and the cells were harvested at 24 hours post transfection.

### Western Blot Analysis

Harvested cells were lysed using radioimmunoprecipitation assay buffer (20 mM Tris-HCl [pH 7.5], 150 mM NaCl, 1% Tween-20, 1% sodium deoxycholate, 1 mM ethylenediaminetetraacetic acid [EDTA], and 5 mM iodoacetoamide [IAA]). The lysates were subjected to sonication, followed by centrifugation at 13,000 × *g* for 5 min to separate the supernatant and pellet. The pellet was resuspended in fresh radioimmunoprecipitation assay buffer buffer. Protein concentrations were determined using the Pierce Bicinchoninic Acid Protein Assay Kit (Pierce, Thermo Fisher Scientific, Inc, Rockford, IL, USA). For western blot analysis, 3 µg of protein per lane were separated using SDS-PAGE and transferred onto polyvinylidene fluoride membranes. Membranes were blocked for 1 hour with 3% bovine serum albumin (BSA) and incubated overnight at 4°C with primary antibodies against GFP, FLAG, and GAPDH (mouse monoclonal; Abcam, Cambridge, UK). Detection was performed using a goat anti-mouse IgG-horseradish peroxidase-conjugated secondary antibody (1:50,000 in 3% BSA blocking buffer; Thermo Fisher Scientific, Cat. #31430) or a goat anti-mouse IgG-alkaline phosphatase-conjugated secondary antibody (1:30,000 in 3% BSA blocking buffer; Sigma-Aldrich, MO, USA, Cat. #A9316).

### Fluorescence Staining for Cellular Imaging

Cells attached to cover glass were transfected with plasmid, followed by the removal of the medium and fixation in methanol at 4°C for 10 minutes. For nuclear staining, cells were treated with 1 μg/mL DAPI (Thermo Fisher Scientific, Cat. #62248) diluted 1/1000 in Dulbecco’s phosphate-buffered saline. Subsequently, the cells were mounted on glass slides using Fluoromount Aqueous Mounting Medium (Sigma-Aldrich, Cat. #F4680). Observations were performed using a Nikon fluorescence microscope (Nikon Instruments Inc., Melville, NY, USA).

### Cell Migration Assay in Transwell Setup

Transfected A549 cells were incubated for 24 hours in fresh DMEM without FBS. Subsequently, the cells were detached using 0.25% trypsin and transferred to a Transwell with a 5-µm pore size (Corning Life Sciences, Corning, NY, USA). The upper surface of the Transwell was precoated with collagen for cell attachment to the upper chamber. To induce cell migration, the lower chamber was filled with DMEM supplemented with 10% FBS. After a 24-h incubation, the migration of living cells was examined by observing the GFP signal using fluorescence microscopy.

### Statistics and Reproducibility

Cell counting was performed in at least 3 independent replicates, and detailed methods and descriptions are provided in the figure legends. Statistical analyses were performed using GraphPad Prism 8.0.1. Statistical significance was verified using 2-tailed Student’s *t*-test. Results were expressed as mean ± standard deviation (SD), and statistical significance was set at *P* < .05.

### *C elegans* Strains

All *C elegans* strains were maintained at 20°C on nematode growth media (NGM) agar plates seeded with *E coli* OP50. The following strains were used in this study: WT Bristol strain N2, PHX9635 *lmn-1(syb9635) I* [*lmn-1(E442C I527C)*], which was generated by Sunybiotech (Fuzhou, China), and IJ2350 *lmn-1(syb9635)*, which was obtained by outcrossing PHX9635 4 times with N2.

### *C elegans* Staining and Microscopy

*Caenorhabditis elegans* staining and microscopy were conducted as previously described (Min et al., 2025). For DAPI staining, L1 synchronized animals were grown on 100 mm NGM plates. At L4 stage, the animals were transferred to NGM plates supplemented with 50 μg/mL 5-fluoro-2′-deoxyuridine (FUDR) (Sigma-Aldrich, Cat. #F0503). At day 4, *C elegans* were harvested and washed with M9 buffer and fixed using 4% formaldehyde (Biotium, CA, USA, Cat. #22023) for 20 minutes. Formaldehyde-fixed animals were then washed 3 times with PBST (0.1% Tween-20) and dehydrated with 70% ethanol overnight at 4°C. Fixed animals were washed twice with PBST and stained with 2 μg/mL DAPI for 15 minutes. The animals were then washed twice with PBST and mounted using VECTASHIELD PLUS Antifade Mounting Medium with DAPI (Vector Laboratories, CA, USA, Cat. #H-2000). Images were obtained using DM6 B microscope equipped with BP 360/40 excitation and LP 425 emission filter set (Leica, Wetzlar, Deutschland). The images were quantified double-blindly. If over two-thirds of nuclei exhibited abnormal morphology, the animals were counted as “High.” If less than one-third of nuclei exhibited abnormal morphology, the animals were counted as “Low.” Otherwise, the animals were counted as “Intermediate.” The circularity of nuclei was measured using ImageJ software (Schneider et al., 2012).

### *C elegans* Lifespan Assay

The lifespan of adult *C elegans* was measured as previously described with minor modifications (Kim et al., 2024, Lee et al., 2024). OP50 *E coli* bacteria were grown in LB containing 10 μg/mL streptomycin at 37°C overnight. Stage-synchronized embryos that were obtained by gravid adults were grown until reaching adulthood at 20°C. Final 5 μM FUDR was spotted on OP50 plates to prevent progeny from hatching. For treatment with NAC (Sigma-Aldrich, Cat. #A9165), NAC was dissolved in double-distilled water, and pH was adjusted to 6.0 using 1 M NaOH. Final 5 mM NAC was additionally spotted on OP50 plates right before transferring animals to prevent rapid oxidization by bacteria (Gusarov et al., 2021). The animals that did not respond to gentle touch with a platinum wire were counted as dead. The animals that burrowed, crawled off the plates, displayed vulval rupture, or internal hatching were censored but included in the statistical analysis. Lifespan assays were performed by 2 independent researchers. Statistical analysis was performed using the online application for the survival analysis portable (OASISp) (Han et al., 2024).

### Measurement of Pharyngeal Pumping and Swimming

The pumping and swimming rate of *C elegans* was measured as previously described (Hwang et al., 2025, Ji et al., 2024, Park et al., 2021). For measuring pumping rates, 10 synchronized animals at days 1, 4, and 7 were assayed for 30 seconds under a dissecting microscope (Zeiss SteREO Discovery V8, Zeiss Corporation, Jena, Deutschland). For measuring swimming rates, 10 synchronized animals at days 1, 4, and 7 were transferred into a well in a 24-well plate containing 1 mL of M9 buffer. The body bending of the animals was recorded using a digital microscope (DIMIS-M, Siwon Optical Technology, Anyang, South Korea) for 30 seconds. The pumping and body bending rates were then converted to number per min.

### Measurement of Total Brood Size

Total brood size was measured as previously described (Park et al., 2021). A single L4-stage animal was transferred onto an NGM plate seeded with OP50 with or without NAC. Each animal was transferred onto a new NGM plate seeded with OP50 with or without NAC every day until the animal stopped laying eggs for 2 consecutive days. Animals that died, exhibited internal hatching, or crawled off before stopping egg-laying were excluded from the analysis.

### Measurement of Developmental Time

Developmental time was measured as previously described (Park et al., 2021). Eggs were obtained from an NGM plate by washing off using M9 buffer. The remaining eggs were incubated at 20°C for 2 hours for hatching. Hatched L1 larvae were then transferred to new NGM plates seeded with OP50 with or without NAC and incubated at 20°C. After 40 hours, the numbers of prefertile adults were counted every 2 hours until all the larvae developed to prefertile adults.

## RESULTS

### R435C Mutation Promotes the Formation of Successive Disulfide Bonds Between the Ig-Like Domains

**Fig. 1:**
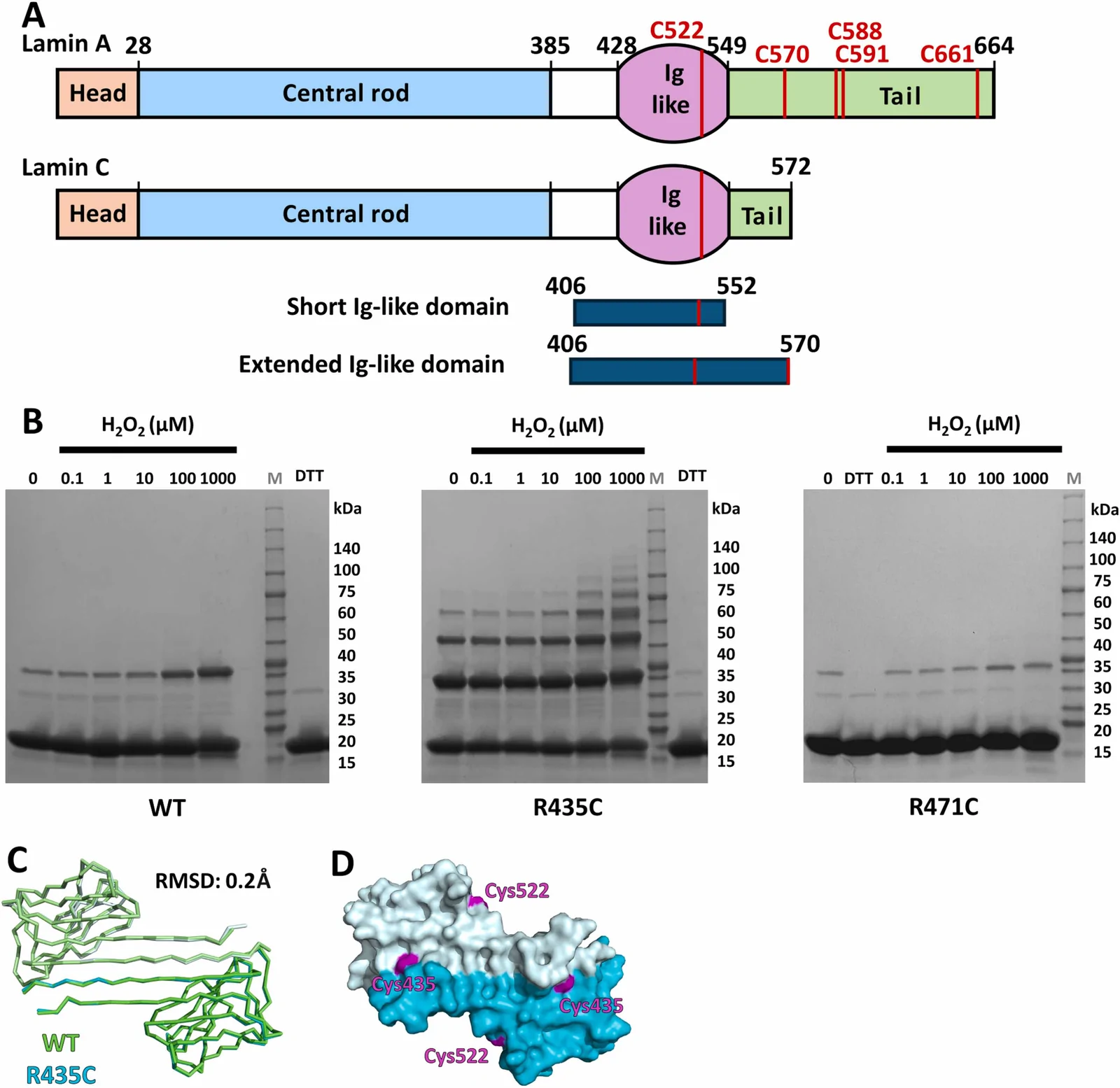
Lamin A and C architecture and the oxidation of the short Ig-like domain proteins. (A) Schematic diagram showing lamin A, lamin C, and their Ig-like domains with cysteine residues used in this study. Cysteine residues within the proteins are indicated with red lines. The Ig-like domain is depicted in 2 variations in this study, with the short form covering residues 406 to 552 and the extended form covering residues 406 to 570. Lamin A features multiple cysteine residues in both the Ig-like domain and C-terminal unstructured region, whereas lamin C has a single cysteine residue in the Ig-like domain owing to alternative splicing resulting in the truncated C-terminal region. (B) SDS-PAGE profiles of the WT (left), R435C (middle), and R471C (right) short Ig-like domains treated with H_2_O_2_. DTT indicates reducing conditions, where 10 mM DTT was added to the samples prior to SDS-PAGE analysis. Each lane includes molecular weight markers (M) for reference, with sizes (kDa) displayed on the right. (C) Ribbon representation of the aligned crystal structures of the WT Ig-like domain (green; PDB code: 7CRG) and R435C mutant (cyan; PDB code: 9UL6). The calculated root mean square deviation (RMSD) between the aligned structures is 0.2 Å, as displayed in the upper-right corner, indicating a high degree of structural similarity. (D) Surface representation of the R435C short Ig-like domain. The 2 chains are distinguished with different shades of cyan (lighter and darker cyan). The mutation-induced Cys435 and original Cys522 residues are highlighted in magenta, indicating their exposure on the protein surface.

To examine the role of the added cysteine residues in the lamin Ig-like domains, we obtained protein fragments corresponding to the Ig-like domains (residues 406-552), harboring each of the point mutations, R435C and R471C, using the *E coli* expression system. The resulting fragments were named short Ig-like domains and terminated upstream of the lamin C C-terminus (residue 572), as shown in Figure 1A.

During the purification of the short Ig-like domain proteins, a notable observation was made for the R435C mutant in nonreducing sodium dodecyl-sulfate polyacrylamide gel electrophoresis (SDS-PAGE) analysis of the aged protein samples after being stored for a few days at 4°C. The gel image showed a ladder-like pattern of protein bands for the R435C mutant protein, whereas this pattern was not observed for the wild-type (WT) or R471C mutant proteins (Fig. S1). The emergence of higher and ladder-like molecular weight bands in the Ig-like domain proteins was attributed to the formation of disulfide bonds, as these bands were absent upon introducing the reducing agent dithiothreitol (DTT). Our findings underscore the role of disulfide bonds in the unique structural manifestations of the R435C mutation in the Ig-like domain.

The formation of disulfide bonds was subsequently monitored through treatment of both mutant and WT Ig-like domain proteins with low levels of H_2_O_2_. After treatment with 0.1 µM H_2_O_2_ for 10 minutes to induce mild oxidative stress, the R435C mutant Ig-like domain fragment exhibited progressive multimer formation, similar to the aged protein sample (Fig. 1B middle). However, the WT and R471C mutant proteins did not exhibit ladder-like patterns under the same experimental conditions (Fig. 1B left and right). Although both the R435C and R471C mutants contained an identical number of cysteine residues, successive disulfide bond formation was not observed in the R471C mutant. Therefore, our findings suggest that the position of the cysteine residue determines sensitivity to disulfide bond formation in the Ig-like domain.

### Crystal Structures of Lamin A/C Ig-Like Domain With R435C Mutation Under Reducing Conditions

To understand the overall structural changes induced by the mutations, we used circular dichroism spectral analysis of the lamin A/C Ig-like domains of the WT and R435C and R471C mutant proteins. The results indicated no substantial differences in the secondary structures of the mutated proteins compared to those of the WT protein (Fig. S2A).

Next, we determined the crystal structure of the R435C mutant Ig-like domain under reducing conditions (PDB code: 9UL6). The structure was nearly indistinguishable from that of its WT counterpart, which has been previously reported (Ahn et al., 2021, Krimm et al., 2002), except for the variation at the mutated 435th residue (Fig. 1C). The thiol group of the native Cys522 is surface-exposed on a beta-strand and forms hydrogen bonds with the backbone carbonyl groups of Glu447 and Glu448 in the adjacent beta-strand (Fig. S2B). The resulting Cys435 was positioned externally with a thiol group on the surface of the Ig-like domain, indicating that the mutant protein was highly sensitive to oxidation (Fig. 1D and S2C). However, as observed in the crystal structures, the distances between the 2 cysteines (Cys522 and Cys435) were not within the distance required to form disulfide bonds (Fig. 1D). Thus, our findings suggest that alternative contacts are required for the formation of disulfide bond-mediated oligomeric states observed in the SDS-PAGE analyses of aged or H_2_O_2_-treated protein samples.

### Linked Bead Pattern of the Oxidized Ig-Like Domains in Electron Microscopy (EM) Images

We further investigated oxidized R435C Ig-like domains after H_2_O_2_ treatment. Protein samples were fractionated based on molecular size using size-exclusion chromatography (SEC) (Fig. S3A). Negative-staining EM of the protein fractions from SEC revealed a bead-like linear oligomeric structure (Fig. S3B). The EM images showed a consistent increase in the length of the linear oligomers, which correlated with the molecular sizes determined using SEC (Fig. S3C).

Because only 2 cysteine residues (Cys435 and Cys522) are present in the short Ig-like domain of the R435C variant, these 2 cysteine residues likely formed continuous disulfide bonds between the Ig-like domains in an intermolecular manner. However, it remained unclear whether these bonds occurred in a head-to-tail manner (Cys435–522) or head-to-head manner (Cys435–435 and Cys522–522). Notably, circular conformations were occasionally observed in some fractions of the EM images (Fig. S3D). These findings provide evidence of the complex oligomerization behavior of the R435C mutants and suggest that the formation of a uniform circular structure may be facilitated by disulfide bonds in the head-to-tail configuration.

### Disulfide-Mediated Oligomers of the R435C Ig-Like Domains in AlphaFold Prediction

To construct molecular models of the oligomeric forms induced by disulfide bonds, the artificial intelligence (AI)-based structural prediction tool AlphaFold2 (Akdel et al., 2022, Jumper et al., 2021, Varadi et al., 2022) was used. Circular forms of the tetramer, pentamer, hexamer, and heptamer of the Ig-like domain were generated using AlphaFold2 prediction (Figs. S4A and D). All models were confirmed to provide high reliability based on the predicted Local Distance Difference Test (pLDDT) and predicted Aligned Error (pAE) metrics, and the hexameric assembly showed the highest scores among the predicted models (Fig. S5). Within these hexameric arrangements, 6 Ig-like domains were positioned in a circular pattern, with Lys467 residues engaging in polar interactions with the Glu448 residues of adjacent protomers (Fig. S4B).

Crucially, the mutated Cys435 residue in one Ig-like domain was positioned to form a disulfide bond with the Cys522 residue from another adjacent chain, facilitating head-to-tail intermolecular bonding within all oligomeric structures in the circular bonding patterns (Fig. S4B). The circular formations, occasionally observed in the EM images, correlated well with the AlphaFold2-predicted model. The predicted molecular contact between the Ig-like domains would be conserved, even if the Ig-like domain forms failed to create a circular arrangement. The oligomers then formed linear structures with a linked bead pattern, as observed by negative-stain EM (Fig. S4C). Based on these findings, we built a molecular model of the linked bead pattern observed in EM.

### Increased Oligomeric Forms in the Extended Ig-Like Domains of R435C and R471C Variants

**Fig. 2:**
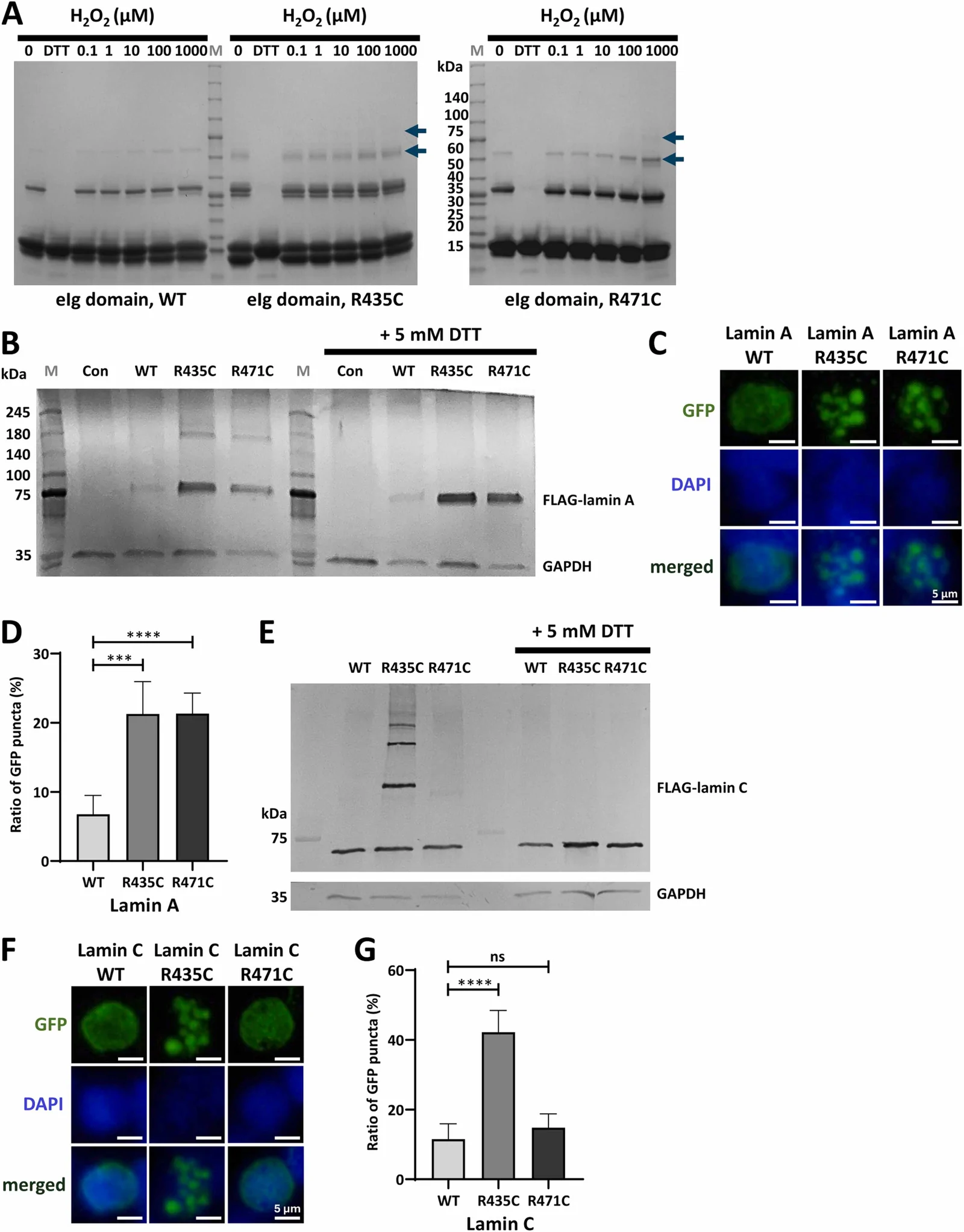
Biochemical and cellular analysis of lamin A and C variants R435C and R471C. (A) SDS-PAGE analysis of the extended Ig-like domain (eIg domain) WT, R435C, and R471C variants after treatment with H_2_O_2_. Lanes labeled with DTT indicate samples that were treated with 10 mM DTT prior to SDS-PAGE analysis. Molecular weight markers (M) are indicated, with sizes (kDa) displayed in the center of the gel. The blue arrows indicate the higher oligomeric bands of the Ig-like domain proteins. (B) Western blot analysis of HEK293E cell lysates overexpressing FLAG-lamin A WT, R435C, and R471C. Proteins were blotted using a FLAG antibody. The “Con” lane represents lysate from cells that were not transfected, whereas the other lanes represent cells expressing the respective lamin A WT or variants. GAPDH was used as a loading control and blotted with GAPDH primary antibody. Molecular weight markers (M) are indicated, with sizes (kDa) displayed. (C) Fluorescence microscopy analysis of GFP-tagged lamin A in HEK293E cells. The nuclear morphology was observed through GFP fluorescence, with representative nuclear shapes shown on the left. Scale bars, 5 μm. Panel D quantifies nuclear morphology abnormalities. The ratio of cells with lamin-GFP punctae to the total number of lamin-GFP-expressing cells was calculated in a 500 × 500-μm area (*N* = 5), with 3 biological repeats performed for each experiment. (E) Western blot analysis was performed on HEK293E cell lysates expressing FLAG-lamin C WT, R435C, and R471C mutants with a FLAG antibody. GAPDH served as a loading control and was detected with a GAPDH primary antibody. Molecular weight markers (M) are displayed in the center of the blot. (F) Fluorescence microscopy images showing the nuclear morphology of GFP-tagged lamin C in HEK293E cells. Representative images are displayed on the left; scale bars, 5 μm. Panel G quantifies nuclear morphology abnormalities by calculating the ratio of cells with lamin-GFP punctae to the total number of lamin-GFP- expressing cells within a 500 × 500-μm area (*N* = 10), based on 3 biological replicates. Statistical significance is indicated by *** for *P* < .001 and **** for *P* < .0001 and “ns” for nonsignificant differences.

To investigate lamin A, we generated an extended Ig-like domain construct (residues 406-570) that explicitly includes the Cys570 residue located within the C-terminal unstructured region. Each variant, including the WT, R435C, and R471C, was treated with H_2_O_2_ and analyzed using SDS-PAGE. The R435C and R471C mutations exhibited higher levels of oligomerization than those observed in the WT, albeit in small amounts (Fig. 2A, blue arrows). These findings suggest that the additional cysteine (Cys570) at the C-terminus of lamin A promotes oligomerization in both mutants, pointing to a shared mechanism underlying progeria.

Notably, the oligomerization pattern of the R435C mutant changed in the extended construct. The ladder-like patterns previously observed in the short Ig-like domain (Lamin C model, Fig. 1B) were abrogated, making the R435C phenotype indistinguishable from that of R471C. Although the recombinant Ig-like domain does not fully mimic endogenous lamin proteins, these results suggest that the cysteine-introducing mutations cause oligomerization in a manner that depends on the position of cysteine and the surrounding cysteine context.

### R435C and R471C Mutations in Lamin A Induce Cellular Aggregation

To assess whether the R435C and R471C mutations induce disulfide-mediated oligomerization within cells, we transiently overexpressed FLAG-tagged full-length lamin A in HEK293E cells. While this transient overexpression does not fully reflect physiological lamin A, it serves as a functional approach to test the aggregation of mutant proteins. Under nonreducing conditions, both R435C and R471C mutations led to increases in oligomerized bands compared to those observed in WT lamin A, although precise comparison was difficult because of higher accumulation of lamin A by the expression of mutated forms than by that of WT (Fig. 2B). This difference may reflect impaired incorporation of mutant lamins into a functional nuclear lamina meshwork and their consequent accumulation as aggregates. Under nonreducing conditions, however, mutant lamin A formed higher-molecular-weight species that were diminished by DTT treatment, supporting the presence of disulfide-dependent oligomerization in cells (Fig. 2B). This observation is consistent with the extended Ig-like domain seen in SDS-PAGE analysis. These findings support the hypothesis that the additional cysteine residues introduced by the R435C and R471C mutations cause disulfide-dependent oligomerization.

To monitor lamin A protein in the cellular nuclear envelope, we transfected green fluorescent protein (GFP)-tagged lamin A containing both mutations into HEK293E cells. The transfected cells showed localized GFP puncta within the nuclear area in both R435C and R471C mutant lamin A, unlike the WT, which exhibited a uniform distribution (Fig. 2C and D). The ratio of cells displaying GFP puncta to the total number of GFP-tagged cells was calculated. The results revealed a considerable increase in puncta formation in R435C and R471C mutant cells compared to those in the WT cells. These findings, combined with the western blotting results, suggest that the GFP puncta observed within the nuclear area were likely caused by disulfide bonds mediated by the additional cysteine residues of the mutations. Therefore, our results suggest that localized clustering of lamin A proteins can result in the nuclear deformation patterns observed in progeroid syndrome.

### R435C Mutation in Lamin C Induces Cellular Aggregation, Unlike R471C

Similar experiments were performed on lamin C, instead of the aforementioned lamin A. To examine the effects of the R435C and R471C mutations in lamin C, we overexpressed the FLAG-tagged variant of lamin C in HEK293E cells. Higher oligomeric forms were observed only in the R435C variant, whereas the WT and R471C variants displayed only monomeric forms (Fig. 2E). This finding is consistent with the ladder-like pattern of the short Ig-like domain with the R435C mutation observed in SDS-PAGE analysis (Fig. 2A).

Next, we overexpressed GFP-tagged lamin C with R435C and R471C mutations in HEK293E cells to investigate the nuclear alterations caused by the mutations. Cells transfected with the R435C variant of lamin C displayed an increased number of localized GFP puncta within the nuclear area, unlike those transfected with the WT or R471C variants (Fig. 2F and G). These results align with the oligomerization patterns of lamin C observed in cell lysates. Our findings indicate that the R435C mutation affects cell types expressing lamin C but not those expressing lamin A. Therefore, further studies are required to investigate the specific role of the R435C mutation in lamin C.

### Diminished Mechanical Stress Resistance in Cellular Nucleus by the R435C Mutation

As aged cells with deformed nuclear envelopes exhibit lower resistance to external mechanical stress (Kang et al., 2024), we measured this mechanical resilience, focusing on the R435C variant. We hereafter specifically focused on the R435C mutation because it consistently induced nuclear defects in both lamin A and lamin C contexts, whereas the R471C mutation did not significantly disrupt nuclear morphology in lamin C-expressing cells (Fig. 2F and G). A Transwell nuclear deformation assay was performed using A549 cells expressing R435C, WT lamin A, and lamin C to test whether the presence of mutant lamin proteins is sufficient to sensitize nuclei to mechanical stress (Fig. S6). This assay forces cells to migrate through narrow porous membranes, imposing mechanical stress on the nucleus. Crucially, cells harboring deformed nuclei failed to withstand this constriction and underwent cell death/rupture during transit. Consequently, the reduced cell count in the lower chamber reflects a loss of viability due to nuclear fragility (Kang et al., 2024).

**Fig. 3:**
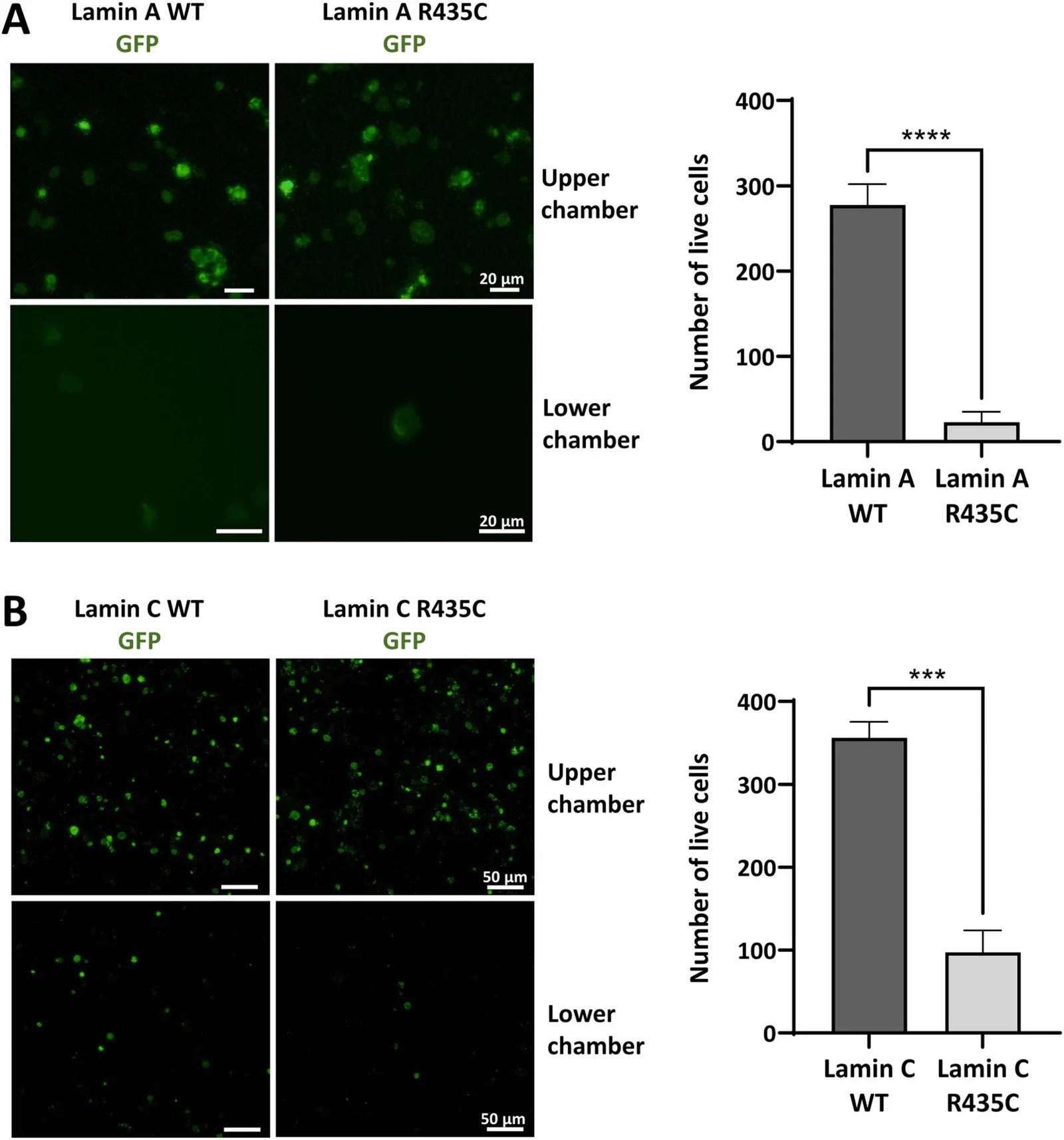
Cell viability analysis of lamin A and C variants using a Transwell migration assay. (A, B) Fluorescence microscopy analysis of GFP-tagged lamin A and C variants in the upper and lower chambers. The analysis shows lamin A WT and the R435C mutant (A), as well as lamin C WT and the R435C mutant (B). The scale bars are shown at the bottom right of each image. Images show the distribution of live cells in the upper and lower chambers. The bar graphs on the right quantify the number of live cells in the lower chamber after migration, revealing a significant decrease in cell migration for both lamin A and lamin C R435C mutants compared to that in their respective WT. **** indicates *P* < .0001 and *** indicates *P* < .001.

Cells transfected with the R435C mutation in both lamin A and lamin C did not survive in the lower chamber of the Transwell plate. By contrast, a significant proportion of WT lamin-transfected cells remained resilient and maintained their viability under the same stress conditions (Fig. 3). Our results indicate that the R435C mutation mechanically weakens the nuclear envelope structure, preventing the passage of cells through the porous membrane without damage. Notably, the R435C mutation causes cellular aging through lamin C, which is rarely involved in cellular senescence.

### Identification of Natural Compounds That Inhibit Disulfide Bond-Mediated Aggregation of Lamin A R435C Mutant

Our results strongly suggest that the harmful effects of the R435C mutation are directly associated with the formation of intermolecular disulfide bonds within both lamin A and lamin C. To address this, we screened natural compounds capable of inhibiting these disulfide bonds in the R435C mutant lamin Ig-like domain. A range of natural compounds, including antioxidants, were tested for their ability to mitigate aggregation in purified short Ig-like domains. SDS-PAGE analysis revealed that sulfur-containing antioxidants, such as GSH and N-acetyl-L-cysteine (NAC), effectively reduced the aggregation of the R435C mutant protein (Fig. S7).

**Fig. 4:**
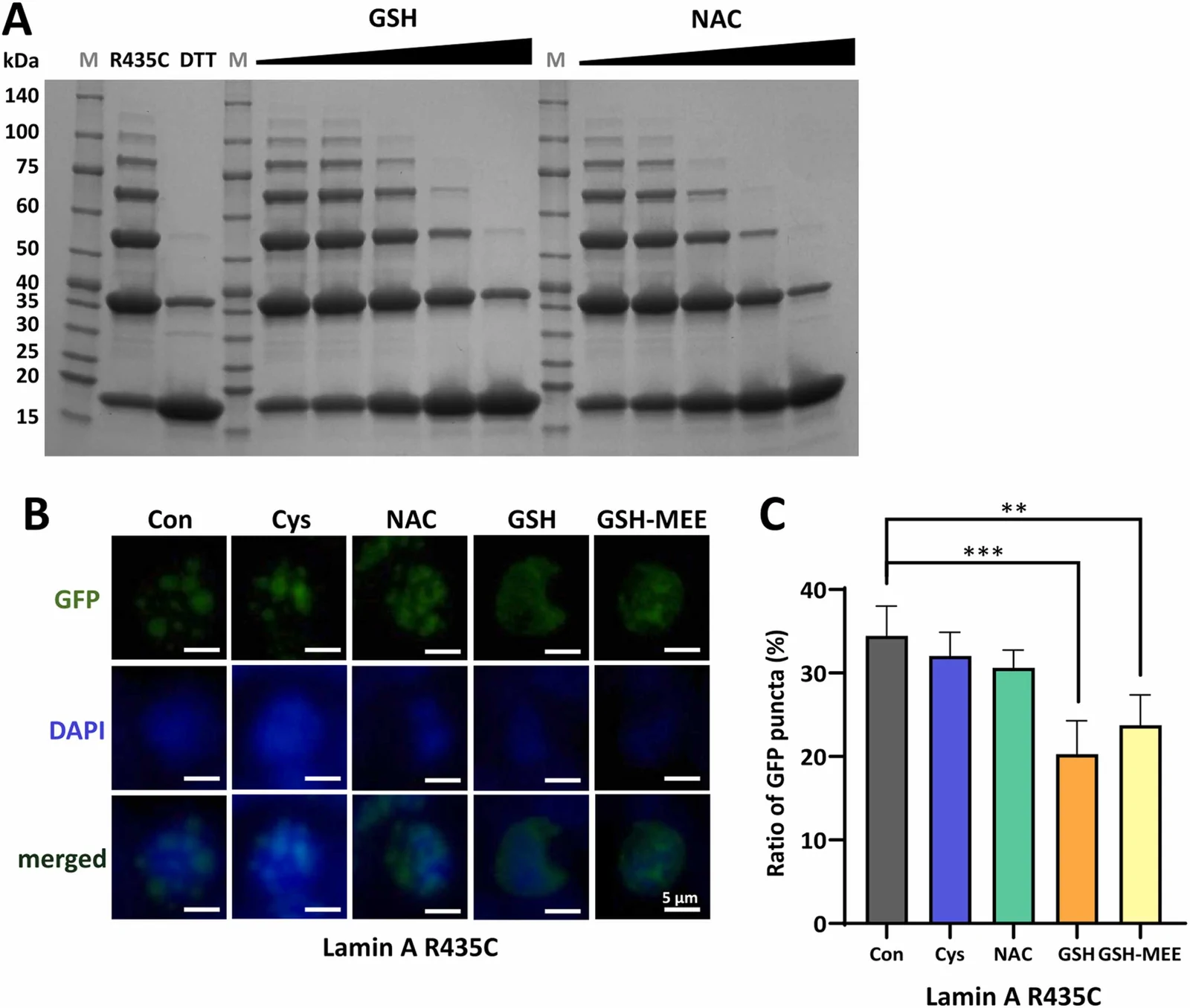
Analysis of oligomerization reduction in lamin R435C by natural compounds. (A) Dose-dependent SDS-PAGE analysis of short Ig-like domain R435C treated with varying concentrations (4, 20, 100, 400, and 2,000 μM) of glutathione (GSH) and N-acetyl L-cysteine (NAC). DTT indicates reduced conditions, where 10 mM DTT was added prior to SDS-PAGE analysis. Molecular weight markers (M) are indicated, with sizes (kDa) displayed on the left. (B) Fluorescence microscopy analysis of HEK293E cells transfected with GFP-lamin A R435C plasmid. Twelve hours post transfection, cells were treated with 1 mM of various natural compounds in DMEM for an additional 12 hours. The final concentration of DMSO in the medium was 1%. Images show GFP fluorescence (green), DAPI staining (blue), and merged views. Scale bar, 5 μm. The conditions are as follows: Con, cysteine (Cys), NAC, GSH, and GSH-monoethyl ester (GSH-MEE). Con indicates no treatment with any compound. (C) Quantification of GFP punctae in treated cells. The ratio of GFP punctae was calculated, with *N* = 5 and 3 independent replicates of each experiment. *** indicates *P* < .001.

Next, we used GSH and NAC with a wide range of doses (ranging from 4 to 2,000 μM) and observed a proportional decrease in protein aggregation, which correlated with the concentration of these sulfur-containing compounds administered (Fig. 4A). To further investigate the effects of these sulfur-containing antioxidant compounds, we treated the HEK293E cells overexpressing the GFP-tagged lamin A R435C variant with GSH and its precursors or derivatives: cysteine, NAC, and GSH monomethyl ether (GSH-MEE). Our results showed that GSH and GSH-MEE significantly inhibited the aggregation of lamin proteins and alleviated nuclear deformation caused by the R435C mutation (Fig. 4B and C). By contrast, cysteine and NAC had a more limited impact on reducing the cellular aggregation of lamin A.

### Cysteine-Engineered *C elegans* LMN-1 Recapitulates Disulfide-Dependent Oligomerization and Nuclear Abnormalities

We used the nematode *C elegans*, an established model organism for aging research (Bong et al., 2026, Kwon et al., 2023, Mack et al., 2018, Tissenbaum, 2015), to investigate whether disulfide bonds in lamin contribute to premature aging by the R453C mutation *in vivo*. Notably, the *C elegans lmn-1* gene lacks cysteine residues, except for the C-terminal CAXX sequence, precluding direct modeling of human lamin A mutations (Fig. S8A). Due to the low sequence homology between human and nematode lamin proteins, conventional sequence alignment methods were inadequate for identifying homologous sites. Instead, we performed a structural alignment using the AlphaFold3-predicted model of the *C elegans* Ig-like domain (Abramson et al., 2024). This approach allowed us to identify Glu442 and Ile527 as putative structural equivalents of human Cys435 and Cys522, respectively (Fig. S8A). By employing CRISPR/Cas9 knock-in technology, we generated a double-substitution mutant of *lmn-1* by simultaneously introducing E442C and I527C. This design was intended to mimic the structural effect of the human R435C mutation (Fig. S8B).

**Fig. 5:**
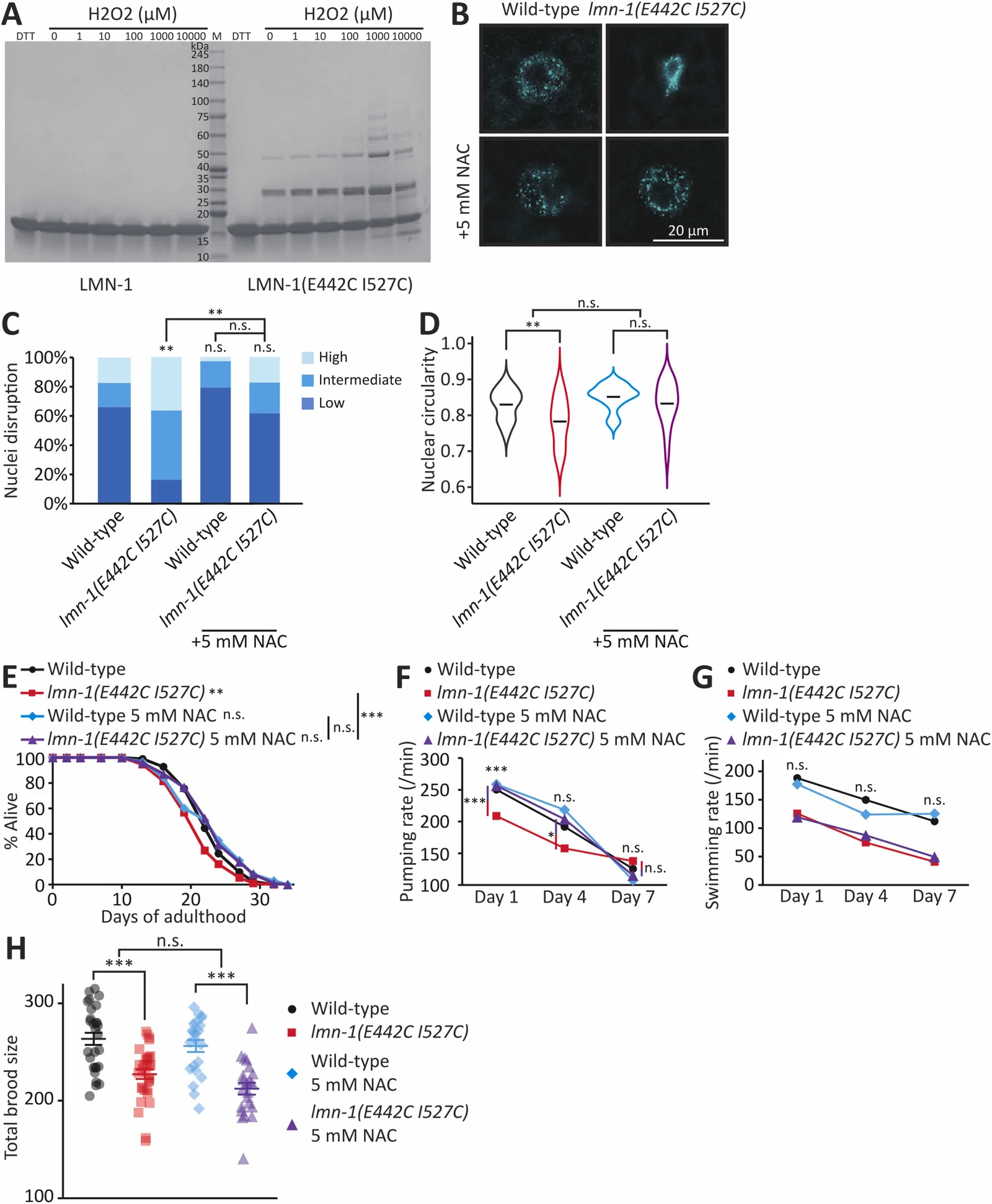
Analysis of nuclear disruption by *lmn-1(E442C, I527C)* mutation in *Caenorhabditis elegans* (Abramson et al., 2024; Schrödinger, 2015) (A) Dose-dependent SDS-PAGE analysis of the short Ig-like domain of LMN-1(E442C, I527C) treated with varying concentrations (1, 10, 100, 100, 1,000, and 10,000 μM) of H_2_O_2_. DTT indicates reduced conditions, where 10 mM DTT was added prior to SDS-PAGE analysis. Molecular weight markers (M) are indicated, with sizes (kDa) displayed on the left. (B) Fluorescence microscopy images of wild-type and *lmn-1(E442C, I527C)* mutant animals with or without NAC stained with DAPI (cyan). Scale bar, 20 μm. (C) Semiquantification of nuclear disruption (N ≥ 30 for each condition), compared to that in the wild-type nuclei, Freeman-Halton extension of the Fisher’s exact test (2-tailed). (D) Circularity of nuclei (*N* ≥ 30 for each condition). (E) Lifespan of wild-type and *lmn-1(E442C I527C)* mutant animals with or without NAC. (F-H) Pharyngeal pumping rates (N = 20 for each condition) (F), swimming rates (*N* = 20 for each condition) (G), total brood size (*N* ≥ 22 for each condition) (H) of wild-type and *lmn-1(E442C I527C)* mutant animals with or without NAC treatment. * indicates *P* < .05, ** indicates *P* < .01, *** indicates *P* < .001, n.s. indicates not significant, log-rank test for E, 2-way ANOVA test, and Tukey’s post hoc test for D and F-H. Interaction *P* values are indicated above data points.

To validate whether these introduced cysteine residues facilitate disulfide bond formation as observed in the human R435C mutant, we purified the recombinant Ig-like domain proteins of *C elegans* LMN-1, encompassing both the wild-type and the double-substitution mutant (E442C I527C). Upon treatment with H_2_O_2_, the mutant LMN-1 protein exhibited a ladder-like oligomerization pattern on SDS-PAGE, mirroring the disulfide bond-mediated aggregation observed in the human Lamin A R435C mutant (Figure 5A). In contrast, the wild-type LMN-1 protein did not show such oligomerization under the same conditions. This biochemical evidence confirms that the engineered cysteines in *C elegans* LMN-1 structurally and functionally recapitulate the pathological disulfide bond formation of human progeria. To assess the impact of these mutations on nuclear architecture, we performed 4′,6-diamidino-2-phenylindole (DAPI) staining on day 4 (middle-aged) adult *C elegans*. We specifically focused on hypodermis, which is a widely accepted tissue for monitoring nuclear morphology due to relatively large and uniform nuclei (Haithcock et al., 2005, Romero-Bueno et al., 2024). We observed that the *lmn-1(E442C I527C)* mutations caused nuclear disruptions within hypodermal cells (Fig. 5B and C).

### NAC Treatment Reverses Disulfide-Associated Nuclear and Aging Phenotypes in *lmn-1(E442C I527C)* Mutants

To test whether the excessive disulfide bond formation is reversible and physiologically relevant, we treated the animals with 5 or 10 mM of NAC, a potent reducing agent and glutathione precursor. We found that 10 mM of NAC treatment decreased the lifespan of WT as previously reported (Gusarov et al., 2021), but 5 mM of NAC only marginally affected the lifespan of WT in our experiments (Fig. S8C). Thus, we performed subsequent experiments using 5 mM of NAC. Unlike in the HEK293E cells overexpressing the GFP-tagged lamin A mutant, the treatment with NAC significantly suppressed nuclear abnormalities in *lmn-1(E442C, I527C)* mutants while having minimal effect on WT animals (Fig. 5B and C). The mutations also led to reduced nuclear circularity, which was partially restored by the NAC treatment (Fig. 5D). These findings suggest that disulfide bonds in LMN-1 accelerate age-dependent nuclear structural disruptions.

We further investigated whether lamin disulfide bonds affect aging by measuring the lifespan of *C elegans* with the *lmn-1(E442C I527C)* mutations. These mutations significantly reduced lifespan, but NAC treatment restored lifespan in the mutants without affecting WT lifespan (Fig. 5E). We also assessed various health parameters, including feeding rate, locomotion (Kim et al., 2024), brood size, and developmental time. The *lmn-1(E442C I527C)* mutations resulted in decreased pharyngeal pumping and swimming rates from day 1 of adulthood, as well as reduced total brood size, indicating premature aging (Fig. 5F-H). NAC treatment partially restored feeding rates in the mutants (Fig. 5F), although it did not improve swimming rates or brood size (Fig. 5G and H). Notably, the *lmn-1(E442C I527C)* mutations did not alter developmental time, regardless of the presence of NAC (Fig. S8D). These results suggest that disulfide bonds in lamin specifically affect aging-related physiology in adulthood. Although these phenotypes do not fully recapitulate human progeroid laminopathy, our findings support a model in which aberrant disulfide bond formation in lamin contributes to nuclear abnormalities and adult physiological defect in *C elegans*, while NAC treatment suggests that aberrant disulfide bonds in lamin are associated with premature aging in adulthood and that targeting these bonds with NAC may offer a potential strategy to reverse age-related phenotypes.

## DISCUSSION

Disulfide bonds play a dual role in protein biology, stabilizing structures or, in some cases, driving pathological aggregation. In this study, we elucidated the molecular mechanisms by which cysteine-containing mutations in lamins A and C contribute to nuclear envelope deformation, a hallmark of cellular aging. By analyzing purified lamin Ig-like domain proteins with varying lengths and cysteine contents, we demonstrated that these mutants form oligomeric structures mediated by aberrant disulfide bonds. This biochemical propensity was translated into cellular aggregation in lamin A- and lamin C-overexpressing cells, underscoring the critical role of disulfide cross-linking in driving nuclear envelope disruption in the presence of mechanical stress. Although a similar propensity for disulfide-mediated cross-linking of lamin was previously reported for the partial lipodystrophy-associated LMNA(R439C) mutation (Verstraeten et al., 2009), our study investigates the progeroid R435C and R471C mutations. Our work also extends these findings by evaluating their physiological impacts at the organismal levels using *C elegans* model.

A noteworthy observation from our biochemical and cellular analyses is the distinct behavior of the R435C and R471C mutations. The R435C mutation led to aggregation and nuclear defects in both lamin C and lamin A, whereas the R471C mutation did so only in lamin A. Because both variants carry an equal number of cysteines, this difference points to the position of the introduced cysteine, rather than its mere presence, as the determinant of cross-linking behavior. Structural modeling suggests that the mutant Cys435 is positioned to form an intermolecular disulfide bond with the native Cys522, a residue present in both isoforms. We emphasize that this specific Cys435-Cys522 pairing is a structural prediction rather than a directly observed bond. Notably, R471C failed to form successive disulfide bonds in the short Ig-like domain even though Cys522 is present there (Fig. 1B), suggesting that Cys471, unlike Cys435, is not favorably positioned to pair with Cys522; instead, R471C appears to depend on the lamin A-specific C-terminal cysteines for cross-linking, consistent with its aggregation being restricted to lamin A. Furthermore, we successfully recapitulated these interactions in an *in vivo C elegans* model. Experimental confirmation that the LMN-1(E442C I527C) mutant protein forms disulfide bonds validates our AlphaFold3 prediction. This demonstrates that the engineered cysteines are in spatially sufficient proximity to interact, resembling the human R435-C522 interface despite the lack of sequence conservation. These findings suggest that disulfide bond formation between lamin proteins, across both lamin isoforms, is a key mediator of premature aging.

We further extended our investigation to the organismal level using the *C elegans* model harboring the corresponding mutations. The purified mutant *C elegans* LMN-1 protein formed disulfide-mediated oligomers under oxidative stress, mirroring the behavior of the human R435C variant. Consequently, the animal model displayed consistent premature aging phenotypes, providing *in vivo* evidence that aberrant disulfide bonds accelerate organismal aging. Importantly, we demonstrated that this pathological process is reversible. Treatment with GSH or its precursors effectively reversed aggregation in purified proteins and cells. Moreover, NAC feeding alleviated nuclear disruptions in progeric *lmn-1* mutant *C elegans*. These results highlight the therapeutic potential of cysteine-based reducing agents to enhance nuclear integrity and delay aging. However, the *lmn-1* mutant *C elegans* should not be viewed as a direct phenocopy of human *LMNA(R435C)*, as mutant animals develop to adulthood, whereas reported severe homozygous *LMNA(R435C)* cases in humans are lethal during infancy (Madej-Pilarczyk et al., 2009). We also did not examine endogenous LMN-1 oligomerization. Furthermore, because we specifically focused on adult aging, we did not perform comprehensive embryonic or gene expression analyses as previously performed using the nematode progeria model (Romero-Bueno et al., 2024). Nevertheless, the progeric *C elegans* strain generated here serves as a valuable *in vivo* platform for further identifying regulators of lamin disulfide cross-linking.

**Fig. 6:**
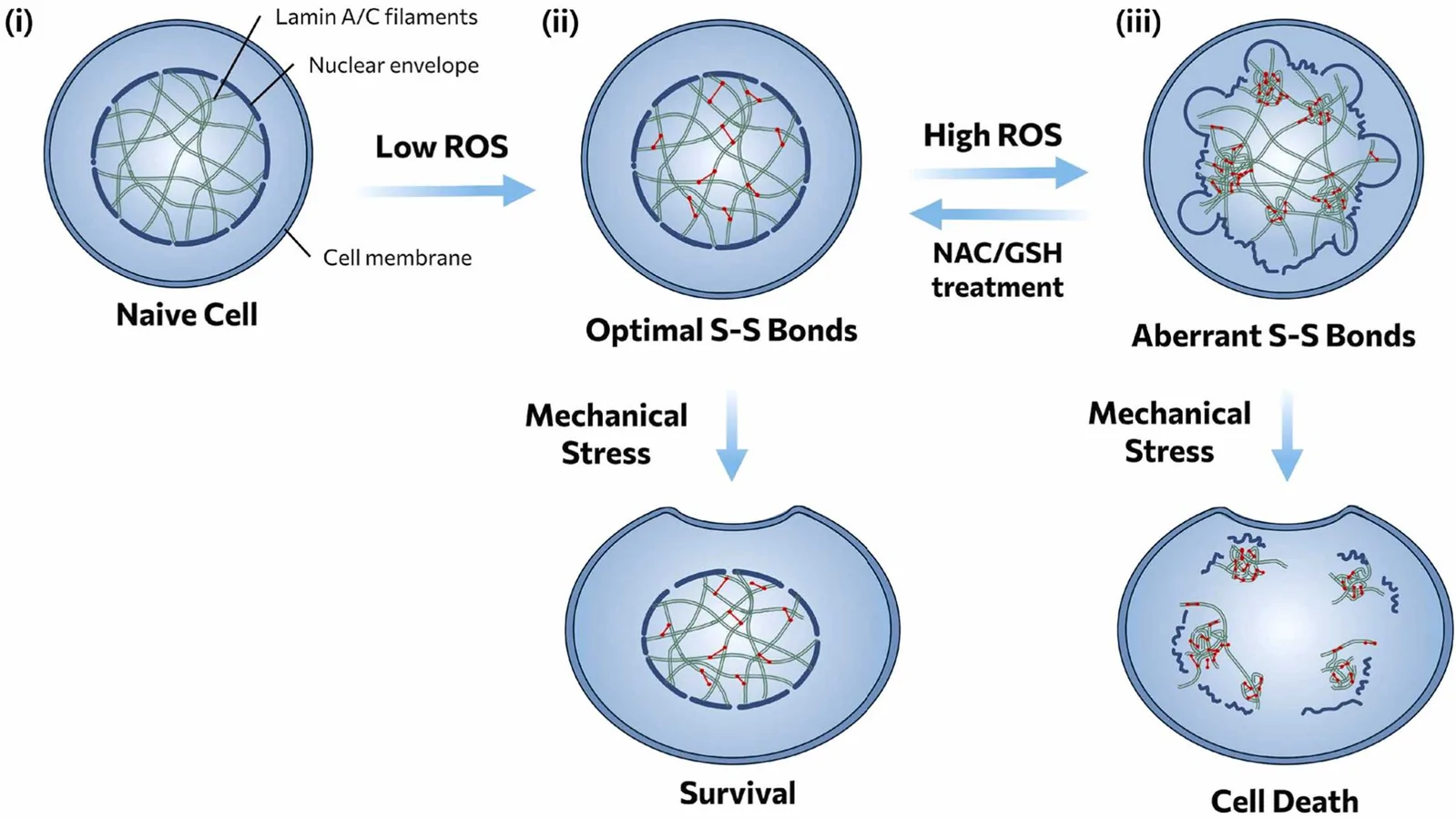
Schematic illustration of the proposed disulfide threshold model. (i) Basal state: In naive cells, lamin filaments remain in a reduced state without reinforcing disulfide bonds. (ii) Moderate reinforcement: Moderate disulfide bonding (red bridges) reinforces the nuclear structural integrity, providing resistance to nuclear rupture under mechanical stress. (iii) Pathological hyper-cross-linking: Excessive cross-linking driven by high reactive oxygen species (ROS) or mutations leads to aberrant aggregation and deformed nuclear structures beyond the “physiological threshold.” This state increases susceptibility to rupture and cell death under mechanical stress. Rescue: This pathological state can be rescued via chemical reduction using NAC or GSH.

The high conservation of cysteine residues in lamin A across vertebrates—typically 5 in humans—raises an intriguing evolutionary question: why are these residues conserved? We propose that disulfide bonds may serve a beneficial role in reinforcing nuclear structure under normal physiological conditions, particularly in tissues exposed to mechanical stress where lamin A is predominantly expressed (Swift et al., 2013). However, we hypothesize a "threshold model": when disulfide cross-linking exceeds a critical level due to mutations or oxidative stress, it leads to irreversible aggregation and structural disruption that overwhelms cellular repair mechanisms (Fig. 6). This nuclear deformation likely contributes to aging by weakening the nucleus's resistance to external mechanical forces. If a deformed nucleus fails to withstand physiological stress—such as muscle contraction or capillary pressure—the genomic DNA becomes vulnerable to damage. Increased susceptibility to mechanical stress can promote DNA double-strand breaks, a primary cause of cellular senescence (Lee et al., 2023, Nader et al., 2021, Park et al., 2024). Thus, a pathway linking excessive disulfide bond formation, nuclear deformation, mechanical stress, and genomic instability emerges as a central mechanism in the aging process.

Our current study has several limitations that need to be addressed in future research. First, while transient overexpression in HEK293E and A549 cells provides evidence that these mutations promote lamin aggregation and reduce mechanical stress resistance, we acknowledge that these cultured cell models do not fully recapitulate the endogenous lamins in patients. Second, although NAC treatment partially suppressed phenotypes in *lmn-1* mutant animals under conditions that did not significantly extend wild-type lifespan, NAC broadly affects cellular redox homeostasis. In addition, intranuclear protein aggregation, such as aggregation of Lmna(H222P), can trigger oxidative stress in cultured cells (Rodriguez et al., 2018). Thus, it remains difficult to fully separate these indirect effects from the direct structural impacts of lamin cross-linking. Therefore, although our biochemical, cell-based, and *C elegans* data support a model in which lamin disulfide bond formation contributes to aging-related phenotypes, further studies using cysteine-to-serine mutants will be needed to determine whether the NAC-mediated rescue is directly attributable to reduced lamin disulfide bond formation. Third, Transwell assay results may have been affected by alternative factors such as decreased motility or chemotaxis rather than mechanical fragility. More specialized assays to assess nuclear integrity in future work will be informative to resolve this issue (Denais et al., 2016, Raab et al., 2016).

In conclusion, our findings provide critical insights into how cysteine-mediated disulfide bonds in lamins contribute to cellular and organismal aging. We highlight the dual nature of lamin cysteines: beneficial for structural integrity up to a threshold, but detrimental when accumulation leads to aggregation. While the precise molecular threshold requires further investigation, our results establish a mechanistic basis for developing therapies targeting redox-dependent cross-linking. Finally, our study offers broader implications for vertebrate aging, suggesting that oxidative stress-mediated changes in lamin architecture can be a conserved mechanism contributing to nuclear deformation.

## CRediT Authorship Contribution Statement

**Seokjun G. Ha:** Writing - review and editing, writing - original draft, methodology, investigation, and formal analysis. **Minho Park:** Writing - review and editing, writing - original draft, investigation, and formal analysis. **Jinwook Lee:** Writing - original draft, investigation, and formal analysis. **Dajeong Bong:** Investigation, formal analysis. **Jinsook Ahn:** Investigation, formal analysis. **Doyeon Kim:** Investigation, formal analysis. **Myung-Ok Kim:** Investigation, formal analysis. **Seung-Jae V. Lee:** Writing - review and editing, supervision, project administration, and funding acquisition. **Nam-Chul Ha:** Writing - review and editing, writing - original draft, supervision, project administration, funding acquisition, and conceptualization.

## Declaration of Competing Interest

The authors declare the following financial interests/personal relationships that may be considered as potential competing interests: Seung-Jae V. Lee and Nam-Chul Ha are editors for Molecules and Cells and were not involved in the editorial review or decision to publish this article. If there are other authors, they declare that they have no known competing financial interests or personal relationships that could have appeared to influence the work reported in this paper.

## Footnotes

Appendix A Supplementary data associated with this article can be found in the online version at doi:10.1016/j.mocell.2026.100389.
