# Supplementary material for: Excessive disulfide bonds in lamin A/C contribute to premature human aging
Source: Mol Cells. 2026 Aug 17;49(10):100389. doi: 10.1016/j.mocell.2026.100389 (PMC13571133; doi:10.1016/j.mocell.2026.100389)
Supplement: Supplementary file 1 [file mmc1.docx]

Excessive disulfide bonds in lamin A/C contribute to premature human aging

Seokjun G. Ha^1^, Minho Park^2^, Jinwook Lee^2,%^, Dajeong Bong^1^, Jinsook Ahn^2,#^, Doyeon Kim^2^, Myung-Ok Kim^2^, Seung-Jae V. Lee ^1,^*, and Nam-Chul Ha^2,^*

^1^Department of Biological Sciences, Korea Advanced institute of Science and Technology, Daejeon 34141, South Korea

^2^Research Institute of Agriculture and Life Sciences, Center for Food and Bioconvergence, Department of Agricultural Biotechnology, CALS, Seoul National University, Seoul 08826, Republic of Korea

^#^Current address: Center for Biomolecular and Cellular Structure, Institute for Basic Science (IBS), Daejeon 34126, Republic of Korea

^%^Current address: Hanmi Pharmaceutical Co., Ltd., Seoul, Republic of Korea

*Corresponding authors: Nam-Chul Ha and Seung-Jae V. Lee

**Email:** hanc210@snu.ac.kr and seungjaevlee@kaist.ac.kr

Keywords: LMNA mutation; immunoglobulin-like domain; progeroid laminopathy; nuclear deformation; oxidative stress

**Supplementary Figure 1. Disulfide-mediated oligomerization of the R435C mutant short Ig-like domain.** SDS-PAGE profile of WT, R435C, and R471C short Ig-like domains. Samples were analyzed under both non-reducing and reducing conditions. Higher molecular weight bands indicate multimer formation owing to disulfide bond formation. Molecular weight markers (M) are indicated, with size (kDa) shown on the left.

**Supplementary Figure 2. Circular dichroism (CD) spectra analysis and local structural environments of wild-type (WT) and mutants (R435C and R471C) short Ig-like domains.** (A) Circular dichroism (CD) spectra of wild-type (WT) and mutant (R435C and R471C) Ig-like domains were measured to assess secondary structure content. The spectra were recorded from 190 to 260 nm. WT is shown in black, R435C in red and R471C in blue. (B) Close-up view of the local environment around Cys522 (Red). The thiol group of Cys522 forms hydrogen bonds with the backbone carbonyl oxygen atoms of Glu447 and Glu448 (Cyan) on the adjacent beta-strand. Yellow dashed lines indicate the distances in Angstroms (Å). (C) Close-up view of the local environment around Cys435 (Red). The side chain of the mutated Cys435 residue is positioned on the surface of the β-strand, with its thiol group exposed to the solvent.

**Supplementary Figure 3. Biochemical analysis of the lamin A/C Ig-like domain with R435C mutation and occasionally observed circular particles in each fraction.** (A) The left panel shows the size-exclusion chromatography (SEC) results for the fractionation of the Ig-like domain. The right panel presents the results of the SDS-PAGE analysis of each fraction. Fractions F3, F6, and F8 are highlighted in red. The molecular weight markers (M) are indicated, with sizes (kDa) displayed on the left. The DTT lane indicates that 10 mM DTT was added to the R435C sample prior to SDS-PAGE analysis. (B) Transmission electron microscopy (TEM) imaging results of DTT, F3, F6, and F8 after negative staining. Scale bar, 50 nm. The red square in the F3 image is magnified in panel D. (C) Measurement of the average particle size observed in each fraction. The number of particles analyzed (N) is DTT: 298, F3: 319, F6: 339, and F8: 323. The average lengths are displayed above each error bar, with units in nanometers (nm). (D) Detailed view of the circular particles observed in each fraction. The red square indicates the magnified area from the F3 image in panel B. The scale bar is displayed at the bottom right and represents 5 nm.

**Supplementary Figure 4. Predicted oligomer structures of the lamin A/C Ig-like domain R435C using Alphafold2 (Jumper et al., 2021).** (A) A cartoon representation of the predicted hexamer structure, with all cysteine residues highlighted in magenta. The dimensions of the measured hexamer model are indicated on the right. (B) Close-up view of the interaction between chains A (green) and B (cyan) in the predicted hexamer structure. All cysteine residues are highlighted in magenta. The black dashed line indicates the ion interaction between Lys467 and Glu448. Cys435 in chain A forms a disulfide bond with Cys522 in chain B. (C) Schematic representation of the expected binding structures of particles observed using TEM. The upper section shows linear particles, whereas the lower section shows circular particles. Actual EM images are displayed on the left, and the predicted oligomerization model of the Ig-like domain is presented on the right. (D) Cartoon representation of various predicted oligomeric forms, including tetramer, pentamer, and heptamer. The two cysteine residues (Cys435 and Cys522) in each chain are highlighted in red.

**Supplementary Figure 5. Analysis of predicted local distance difference test (pLDDT) and predicted aligned error (PAE) for the short Ig-like domain, exerted by Alphafold2 (Jumper et al., 2021).** (A) The predicted local distance difference test (pLDDT) scores for the Ig-like domain residues. Each point represents a residue index, with color indicating the confidence score from blue (high confidence) to red (low confidence). The high pLDDT scores suggest well-defined local structures. (B) The predicted aligned error (PAE) matrix for the Ig-like domain. The matrix shows the predicted alignment error between residues, with colors representing the error magnitude from yellow (low error) to purple (high error). The low PAE values along the diagonal indicate high confidence in the alignment of the corresponding regions.

**Supplementary Figure 6. Schematic Diagram of Transwell Migration Assay for Lamin A/C Variant-Expressing Cells through a Collagen-Coated 5 μm Pore Membrane.** Cells expressing lamin A or lamin C variants are seeded in the upper chamber, which is separated from the lower chamber by a collagen-coated membrane with 5 μm pores (Corning Life Sciences, Corning, NY, USA). This setup allows for the assessment of cell migration through the pores.

**Supplementary Figure 7. Screening of natural compounds for the reduction of disulfide-mediated oligomerization in the R435C mutant.** SDS-PAGE analysis of short Ig-like domain R435C treated with 1 mM of various natural compounds (including DTT) at 50 μM concentration, incubated at 37°C for 12 hours. The reaction was terminated by adding 2.5 mM IAA. Compounds were dissolved in DMSO, with a final DMSO concentration of 2%. Each lane represents the following treatments, 1: DMSO (con), 2: DTT, 3: GSH, 4: garlic extract, 5: N-acetyl L-cysteine, 6: vitamin C, 7: vitamin E, 8: β-carotene, 9: morin, 10: quercetin, 11: fisetin, 12: resveratrol, 13: curcumin. Molecular weight markers (M) are indicated, with sizes (kDa) displayed on the left. Compounds that significantly reduced oligomerization are highlighted in red: 3: GSH, 5: N-acetyl L-cysteine.

**Supplementary Figure 8. Sequence alignment and structural analysis of *C. elegans* LMN-1 and human lamin A and the effects of NAC on lifespan.** (A) The full amino acid sequences of C. elegans LMN-1 and human lamin A are aligned by ClustalX (Larkin et al., 2007). Key functional domains are indicated: the Ig-like domain (blue bar) and the C-terminal tail region (red bar). The Zmpste24 cleavage site and the farnesylation site of human lamin A are indicated by the green bar and orange arrow, respectively. Putative structural equivalents – Glu442 and Ile527 in LMN-1 for human lamin A residue Arg435 (cyan arrow) and Cys522 (yellow arrow) – are highlighted within red box. (B) Cartoon representation of aligned structures of the *C. elegans* LMN-1 (blue) and human lamin A (green; PDB code: 7CRG) by PYMOL (Schrodinger, 2015). Structure of *C. elegans* LMN-1 is predicted by Alphafold3 (Abramson et al., 2024). E442 and I527 in LMN-1 structurally correspond to R435 and C522 in lamin A, respectively. (C) Lifespan of wild-type animals treated with 5 or 10 mM of NAC. (D) Developmental time to adulthood of wild-type and *lmn-1(E442C I527C)* mutant animals with or without NAC treatment. *** indicates p < 0.001, n.s. indicates not significant, log-rank test for (C), two-way ANOVA for (D).

**Supplementary Table. 1 X-ray diffraction and refinement data**

|  | **Lamin A/C Ig-like domain R435C** |
| --- | --- |
| **Data collection** |  |
| Beamline | PAL 5C |
| Wavelength (Å) | 0.9795 |
| Space group | *P*6_4_22 |
| Cell dimensions |  |
| *a, b, c* (Å) | 92.0, 92.0, 204.8 |
| Resolution (Å) | 50.0-2.50 (2.54-2.50) |
| *R*_pim_ | 0.019 (0.070) |
| *R*_merge_ | 0.090 (0.351) |
| *I/σI* | 34.1 (11.5) |
| Completeness (%) | 94.8 (95.6) |
| Redundancy | 17.6 (18.2) |
| **Refinement** |  |
| Resolution (Å) | 34.41-2.50 |
| No. of reflections | 17125 |
| R_work_/R_free_ | 0.203/0.276 |
| No. of total atoms | 3039 |
| Wilson B-factor (Å^2^) | 35.9 |
| RMSD. |  |
| Bond lengths (Å) | 0.008 |
| Bond angles (°) | 1.000 |
| Ramachandran plot |  |
| Favored (%) | 97.00 |
| Outliers (%) | 0.00 |
| PDB code | 9UL6 |
| * The values in parentheses are for the highest resolution shell. | |

**REFERENCES**

Abramson, J., Adler, J., Dunger, J., Evans, R., Green, T., Pritzel, A., . . . Jumper, J. M. (2024). Accurate structure prediction of biomolecular interactions with AlphaFold 3. *Nature, 630*(8016), 493-500. doi:10.1038/s41586-024-07487-w

Jumper, J., Evans, R., Pritzel, A., Green, T., Figurnov, M., Ronneberger, O., . . . Potapenko, A. (2021). Highly accurate protein structure prediction with AlphaFold. *Nature, 596*(7873), 583-589.

Larkin, M. A., Blackshields, G., Brown, N. P., Chenna, R., McGettigan, P. A., McWilliam, H., . . . Lopez, R. (2007). Clustal W and Clustal X version 2.0. *bioinformatics, 23*(21), 2947-2948.

Schrodinger, LLC. (2015). *The PyMOL Molecular Graphics System, Version 1.8*.
